# A Multidisciplinary Approach to Malocclusion Caused by Facial Multiple Fracture

**DOI:** 10.1155/2022/5209667

**Published:** 2022-03-03

**Authors:** Daishi Arai, Akihiro Yasue, Shinya Horiuchi, Eiji Tanaka

**Affiliations:** ^1^Department of Orthodontics and Dentofacial Orthopedics, Tokushima University Graduate School of Oral Sciences, Tokushima, Japan; ^2^Department of Orthodontics and Dentofacial Orthopedics, Tokushima University Graduate School of Biomedical Sciences, Tokushima, Japan; ^3^Department of Orthodontics and Dentofacial Orthopedics, Tokushima University Hospital, Tokushima, Japan

## Abstract

In the case of multiple facial fractures, a simple open reduction occasionally causes various disorders during healing process after the surgery. Moreover, esthetic disturbance of a facial deformity might be induced. Therefore, the acquisition of facial symmetry and the recovery of occlusal and masticatory functions become increasingly important. This case report presents a successful treatment of facial multiple fracture induced by a car accident. A 20-year-old male was diagnosed with suffered multiple midface and mandibular fractures induced by a car accident. Midface fractures included the LeFort I and II type fractures, as well as sagittal fracture at midline and fractures from right maxillary sinus anterior wall to orbital wall. In the mandible, midline and left body fractures were detected. The patient underwent open reduction and rigid fixation of the fractured left zygoma, comminuted LeFort I and II fractures, and midline and left body of the mandible with intermaxillary fixation by multibracket appliance; maxillary osteotomy with iliac bone grafting; orthognathic two-jaw surgery with coronoid process grafts onto the depressed zygoma; and onlay graft of hydroxyapatite block on mandible. As the result, the multidisciplinary treatments successfully recover functions and esthetics to the satisfactory level of the patient with multiple facial fractures. As treatments for multiple facial fractures are required complexity due to the extent of trauma, multidisciplinary approach under the close cooperation between hospital departments is thought to be important.

## 1. Introduction

Patients involved in motor vehicle accidents often suffer from multiple midface and mandibular fractures including the damage of both hard and soft tissues [1]. In the case of multiple facial fractures involving maxilla, mandible, and zygomatic bone, a simple open reduction occasionally causes various disorders during healing process after the surgery such as trismus, masticatory disturbance, swelling, and postoperative morbidity, leading to a decrease of a patient's quality of life [2]. Moreover, esthetic disturbance of a facial deformity might be induced. These sequelae are often caused by the malunion of fracture sites, and long-term treatment is needed in some cases. Therefore, the acquisition of facial symmetry and the recovery of occlusal and masticatory functions become increasingly important. Even if the operation for the purpose of functional and esthetic recovery was executed, refracture of malunion, scar tissue dissection, and subsequent bone mass insufficiency and dead space may cause another malunion. Hence, multilateral diagnoses and advanced treatments are required for these improvements.

This article demonstrates a clinical experience of multidisciplinary approach to oral and esthetic rehabilitation from maxillofacial fracture induced by a car accident.

## 2. Case Presentation

**Figure 1:**
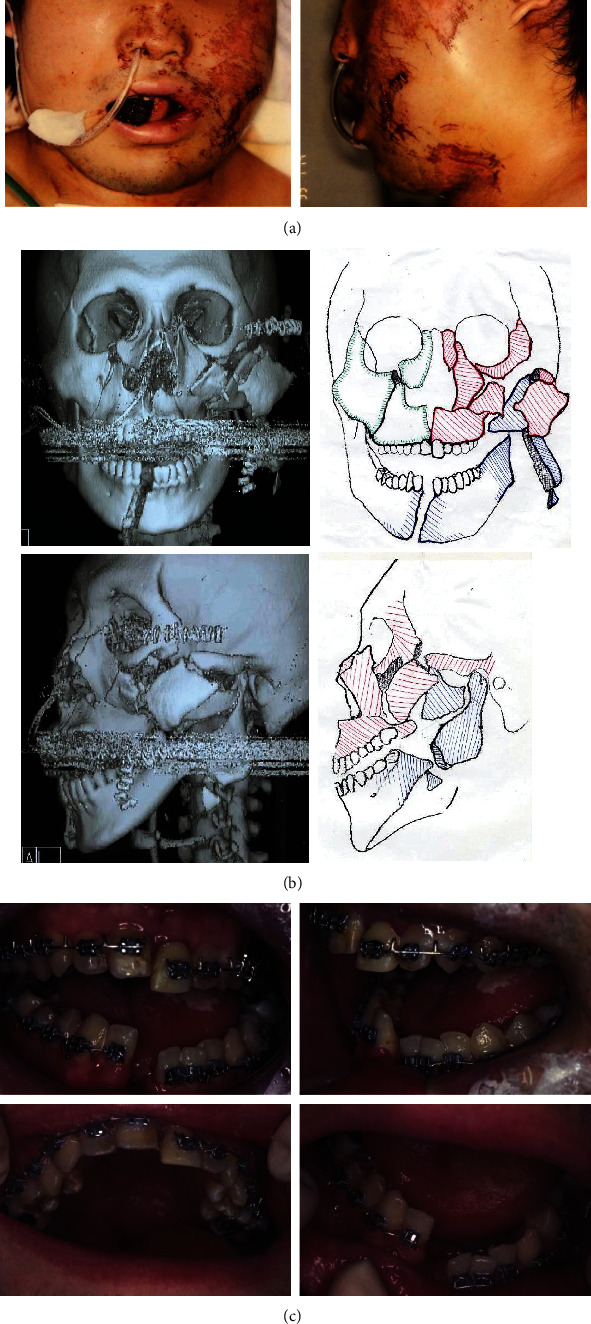
Facial photographs (a) and cephalic CT images with schematic illustrations immediate after injury (b) and intraoral photographs taken after placement of surgical braces for the intermaxillary fixation (c).

A 20-year-old male was first sent to an emergency hospital after a car accident. His maxillofacial injuries had included dehiscence wounds on the left temporal region, right frontal region, left lower eyelid, and buccal region (Figure 1(a)). Furthermore, as arterial bleeding from the nose and mouth was observed, wound suturing and astriction were then performed. Symptoms of cranial nerves were not confirmed regardless of the clouding of his consciousness. He had his normal eyesight, and abnormal findings were not observed even in cephalic computed tomography (CT) images. Fracture of fifth rib was found by chest radiography. On the other hand, tracheotomy had been performed to avoid suffocation due to airway constriction caused by severe swelling and subcutaneous bleeding from the left cranium to submandibular region. Four days after injury, his physical status was recovered, then he was transferred to the Tokushima University Hospital for close inspection and medical treatment of gnathic bone fractures.

From the cephalic CT images, midface fractures included fractures of the Le Fort I and II planes, as well as sagittal fracture at midline and fractures from right maxillary sinus anterior wall to orbital wall (Figure 1(b)). In the mandible, the body was completely separated off at midline, distal region of the left third molar, coronoid process, and condylar process. The fractured end of condylar process was displaced to the outer side. As in the intraoral findings, the laceration on the fracture line at mandibular incisor area and the submucous bloody spots on left sublingual region were observed, the continuous dental arch was lost with mobility, and the cross bite on the left molar region and anterior open bite were observed (Figure 1(c)). A clinical diagnosis was made of malocclusion due to facial multiple fractures, and the following treatment plans were determined: (1) closed reduction by intermaxillary elastics, (2) open reduction, and (3) intermaxillary fixation using a multibracket appliance. The patient gave written informed consent before beginning treatment.

**Figure 2:**
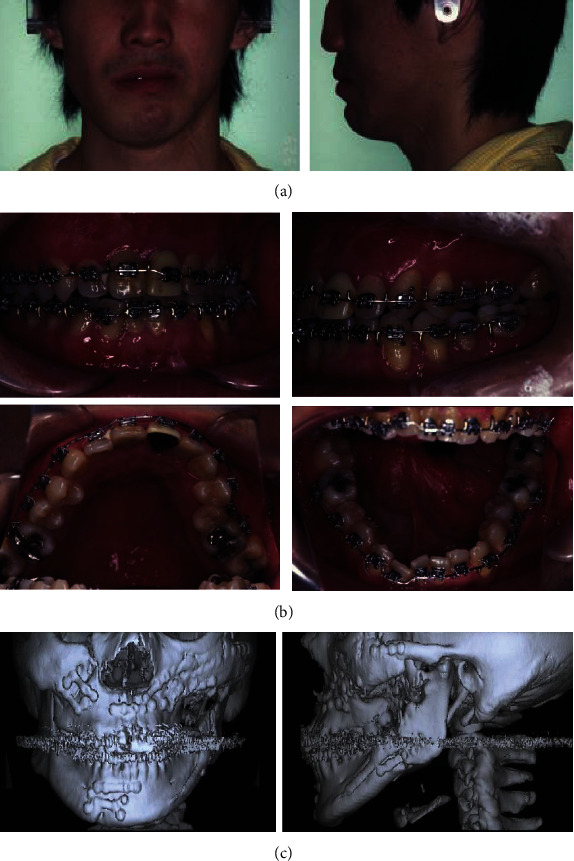
Facial (a) and intraoral photographs after 9 months (b) and cephalic CT images after open reduction (c).

**Figure 3:**
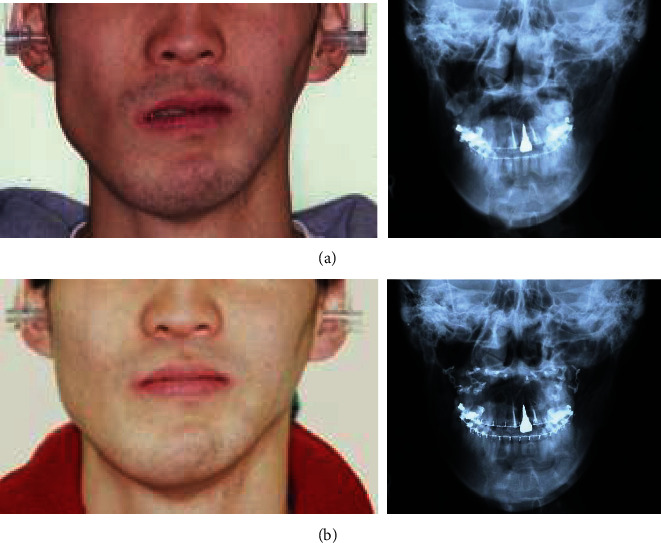
Facial photographs and frontal cephalograms before (a) and after two-jaw surgery (b).

**Figure 4:**
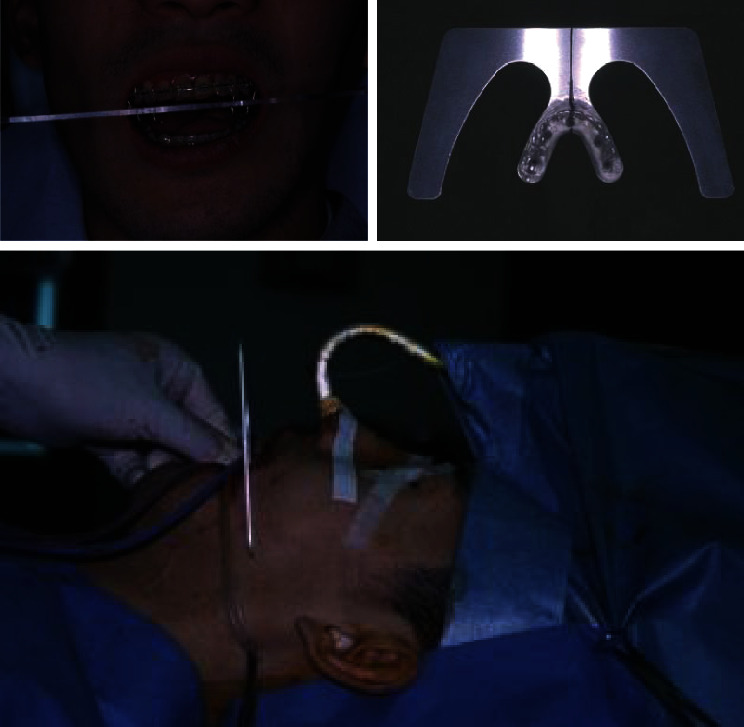
Maxillary positioning using an occlusal plane table as a parallel indicator to Frankfort horizontal plane.

**Figure 5:**
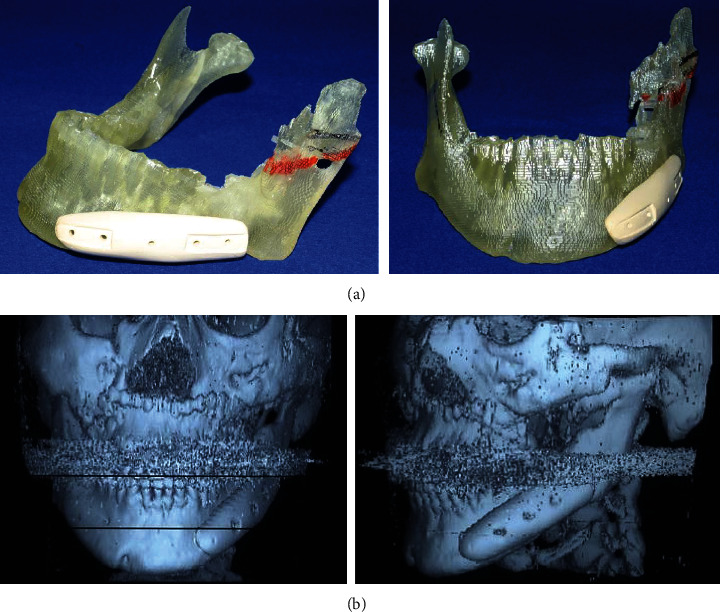
HAP-TCP blocks for onlay grafting on three-dimensional model (a) and cephalic CT images after onlay grafting of HAP-TCP block (b).

**Figure 6:**
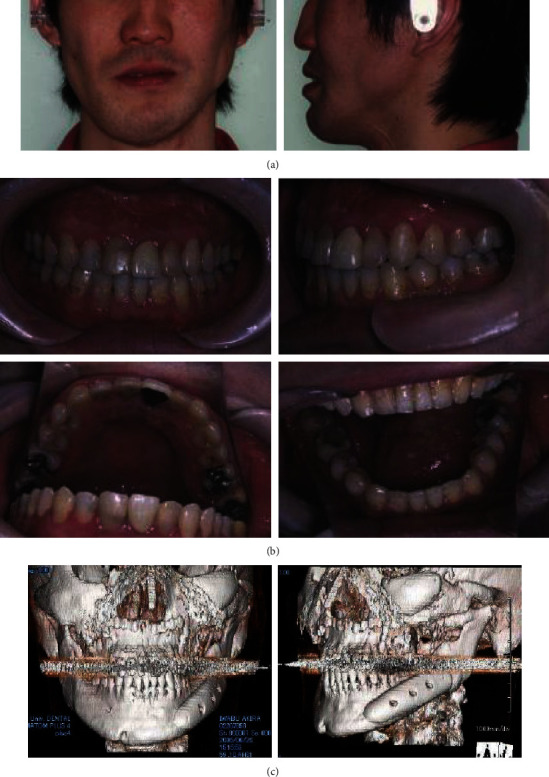
Facial (a) and intraoral photographs (b) and CT images after postoperative orthodontic treatment (c).

To tract both dentitions and maxilla, the intermaxillary elastics and the maxillary protraction headgear were started. The fractures were operated on open reduction one week after closed reduction. The mandible with a fewer fracture was first reduced and fixed temporarily to establish a yardstick for the occlusal plane that the original position is unclear, then shifted to the reduction and fixation of the maxillary and zygomatic bones. After that, the mandible was reduced again following to a plaster model reconstructed as a prospective occlusion. The operation was completed with intermaxillary fixation. After 31 days of internal fixation, postsurgical orthodontic treatment was executed for 9 months. Maximum mouth opening without pain was 30 mm within 3 months after surgery; meanwhile, the scar revision surgery on the left buccal region and the correction of the left lower eye lid ectropion were performed by plastic surgeons. The submucous resection of nasal septum by otolaryngologists was also undergone. Although almost stable and functional occlusion was obtained, asymmetric frontal view caused by occlusal cant in the frontal plane was remained (Figures 2(a) and 2(b)). At the same time, upper left maxillary osteotomy with iliac bone grafting was executed as the left molar crossbite was impossible to improve with tooth movement alone (Figure 2(c)). At this time, the patient reported to be fairly satisfied with pronouncing words and eating foods; however, he strongly requested the improvement of his facial asymmetry. Then, two-jaw surgery was decided to undergo with onlay grafting of right coronoid process onto the left side of depressed buccal area. The application of hydroxyapatite blocks for skeletal reconstruction was explained to the patient in the case that the asymmetry still remained after surgery. Before surgery, the blood flow changes due to injury were suspected and examined by angiography. Then, it was ascertained that the blood flow where the left external carotid artery branches off into the lingual, facial, and maxillary artery was not identified. Next, preoperative three-dimensional model was prepared to understand the accurate prediction. As a result, Le Fort I osteotomy was adopted for the modification of the maxillary cant. As to mandible, bilateral intraoral vertical ramus osteotomy (IVRO) was chosen as sagittal split ramus osteotomy (SSRO) was estimated to be difficult because of malunion from the former open reduction. Another reason was to decrease the bleeding risk from scar tissue. After 1 year and 5 months of preorthodontic treatment, two-jaw surgery was executed (Figure 3(a)). As a surgical procedure, an occlusal plane table was used as a parallel indicator to Frankfort horizontal plane for fixing maxilla (Figure 4). Then, IVRO was performed bilaterally. Confirming 40 mm of maximum jaw opening during operation, the bone fragment removed from right coronoid process was shaped into 3 × 2 cm block for onlay grafting in the depression of left buccal region. After two-jaw surgery, although the shifted chin point and occlusal cant were improved, the patient still complained the meagerness of his left mandibular angle region (Figure 3(b)), then decided to undergo another onlay graft of hydroxyapatite-tricalcium phosphate composite (HAP-TCP) blocks (Figures 5(a) and 5(b)) and free vascularized fat graft following patient's wishes. At the end of postoperative treatment, facial asymmetry was improved to his satisfactory level, and an acceptable occlusion with adequate interincisal relationship was obtained (Figures 6(a) and 6(b)). From CT images, bone healing was completed (Figure 6(c)).

## 3. Discussion

Jaw deformity could be induced by not only acquired skeletal deformation but also malunion after traumatic bone fractures. For these patients, comprehensive treatment involving multiple disciplines is needed to successfully restore esthetics and functions and allow the patient to regain their self-esteem; on the other hand, several points such as soft tissue injury and displacement of bone fragment should be considered in orthognathic surgery. For example, bleeding risk increases. There are not a few cases of anatomical change in blood streams in scar tissue caused by injury. In this case, blood streams in the site of injury were examined before surgery by angiography to choose the safer and more reliable operative method. On the other hand, blood storage by autotransfusion was prepared in advance.

Since surgical movements of the jaws are complicated three-dimensional shifts of geometrically complex structures, the establishment of a yardstick for osteotomy is serious. An intermediate splint using the mandible to place the maxilla is generally adopted to reach the predictable destination in bimaxillary orthognathic surgery [3]. The splint was generally fabricated on the articulator to relate the repositioned maxillary cast to the preoperative mandibular cast before surgery. In the surgery, after maxilla is fixed to the mandible with the intermediate splint interposed, vertical dimension was determined by external reference measure to avoid the accidental error. A vertical measurement is recorded from the medial palpebral fissure to the maxillary orthodontic arch wire between incisors as it is a very stable reference point which is easily accessed pre- and intraoperatively [4]. Moreover, it eliminates the need for placement of a wire or a screw at nasion and the possible soft tissue scarring. This external vertical measurement is about as accurate as the placed rigid fixation [5, 6], and is useful in all Le Fort I maxillary osteotomies. In this case, medial palpebral fissure is used as a standard to avoid scarring soft tissue.

Obtaining prearranged occlusal plane angle is also one of the most important points for acquisition of a good treatment outcome. A splint combined with occlusal plane table was used to determine occlusal plane in this case. Thus far, the following indices have been used for occlusal plane alteration, such as the angular relationship between the occlusal and Frankfort horizontal plane [7–9], the facial appearance in harmony with the chin, upper lip position, and lower facial height by the rotation of the maxilla-mandible complex around ANS, point A, and the incisal edge of maxillary central incisor [10, 11], and parallel to naso-auditory meatus line. In this case, the confirmation of occlusal plane was easily achieved anteroposteriorly with the occlusal plane table equipped to the splint established parallel to Frankfort horizontal plane. As was estimated before surgery, postsurgical occlusal plane angle was decreased 4.2°. The improvement of asymmetry was also verified by occlusal plane table. For the procedure of osteotomy, SSRO seemed to be difficult because of the malunion of mandible, and to minimize bleeding from scar tissue and operative duration, IVRO was executed.

Facial asymmetry still kept the patient feel dissatisfaction even if occlusal function was recovered. After the two-jaw surgery, although bone grafting from right coronoid process to left depressed cheek area was performed, patient insisted the enlargement of left mandibular body. As the modification of the mandibular asymmetry required huge bone mass, an artificial bone grafting with free vascularized fat grafting from femur was chosen for the augmentation. Many studies have confirmed the osteoconductivity of composite biomaterials. Gosain et al. [12] compared volume maintenance of onlay grafts and bone replacement of implants in sheep among hydroxyapatite ceramic, HA/TCP, and hydroxyapatite cement past. The HA/TCP, then, demonstrated good scores in both maintenance and replacement [12]. We also used hydroxyapatite/beta-tricalcium phosphate (HA/*β*-TCP; NGK SPARK PLUG CO., LTD., Japan), which is a ceramic composed of a combination of hydroxyapatite [Ca_10_(PO_4_)_6_(OH)_2_] and beta-tricalcium phosphate [Ca_3_(PO_4_)_2_], heated at 900 to 1300 degree Celsius and has a porous structure including large pores. Symmetrical mandible is still retained 1 year and 4 months after grafting, and the HA/TCP efficacy was confirmed. It was reported that the transplants of autologous culture-expanded bone marrow stromal cells (BMSCs) combined with HA/TCP scaffolds formed cortico-cancellous bone in dog craniofacial skeleton and showed long-term stability; meanwhile, HA/TCP particles alone underwent a high degree of resorption [13]. Long-term careful observation is then required for the patient. Recently, the use of polylactic acid/polyglycolic acid (PLA/PGA) biodegradable membranes in the form of volumetric chambers to carry an underlying HA/TCP onlay graft was also reported [14]. PLA/PGA is already known as biocompatible, geometrically stable, and favourably degradable product. Almasri et al. showed its great osteoconductivity of the distribution of bone arranged in multiple semicircular deposits and significant bony surface area by onlay graft in rabbit, indicating that this method would be an excellent option to overcome disadvantages of autogenous bone grafts, such as morbidity at donor site, limited quantity, and rapid resorption when compared with nonautogenous grafts.

The final results with regard to facial contour, maxillomandibular relationship, and symmetrical feature were satisfactory for the patient, which indicates the importance of multidisciplinary treatment for the correction of malocclusion due to maxillofacial bone deformities from fractures.

## 4. Conclusion

The patient with deformities of the maxillofacial bones that were caused by facial multiple fractures are reported. Multidisciplinary treatment was successfully used to achieve cosmetic reconstruction and recovery of maxillomandibular function and occlusion for the patient. In this case, the esthetics and functions were difficult to recover only with the first open reduction, and then, the secondary correspondence was needed. As treatments for multiple facial fractures are required complexity due to the extent of trauma, multidisciplinary approach under the close cooperation between hospital departments is thought to be important.

## References

[1] Chuang K. T., Hsieh F., Liao H. T. (2019). The correlation of age and patterns of maxillofacial bone fractures and severity of associated injuries caused by motorcycle accidents. *Annals of Plastic Surgery*.

[2] Dongol A., Jaisani M. R., Pradhan L., Dulal S., Sagtani A. (2015). A randomized clinical trial of the effects of submucosal dexamethasone after surgery for mandibular fractures. *Journal of Oral and Maxillofacial Surgery*.

[3] Ellis E. (1999). Bimaxillary surgery using an intermediate splint to position the maxilla. *Journal of Oral and Maxillofacial Surgery*.

[4] Lapp T. H. (1999). Bimaxillary surgery without the use of an intermediate splint to position the maxilla. *Journal of Oral and Maxillofacial Surgery*.

[5] Cope M. R. (1994). Measuring changes in maxillary height during osteotomy surgery. *The British Journal of Oral & Maxillofacial Surgery*.

[6] Ferguson J. W., Luyk N. H. (1992). Control of vertical dimension during maxillary orthognathic surgery. *Journal of Cranio-Maxillo-Facial Surgery*.

[7] Wolford L. M., Chemello P. D., Hilliard F. W. (1993). Occlusal plane alteration in orthognathic surgery. *Journal of Oral and Maxillofacial Surgery*.

[8] Wolford L. M., Chemello P. D., Hilliard F. (1994). Occlusal plane alteration in orthognathic surgery--part I: effects on function and esthetics. *American Journal of Orthodontics and Dentofacial Orthopedics*.

[9] Chemello P. D., Wolford L. M., Buschang P. H. (1994). Occlusal plane alteration in orthognathic surgery--part II: long-term stability of results. *American Journal of Orthodontics and Dentofacial Orthopedics*.

[10] McCollum A. G., Reyneke J. P., Wolford L. M. (1989). An alternative for the correction of the class II low mandibular plane angle. *Oral Surgery, Oral Medicine, and Oral Pathology*.

[11] Sarver D. M., Weissman S. M., Johnston M. W. (1993). Diagnosis and treatment planning of hypodivergent skeletal pattern with clockwise occlusal plane rotation. *The International Journal of Adult Orthodontics and Orthognathic Surgery*.

[12] Gosain A. K., Riordan P. A., Song L. (2005). A 1-year study of hydroxyapatite-derived biomaterials in an adult sheep model: III. Comparison with Autogenous Bone graft for facial augmentation. *Plastic and Reconstructive Surgery*.

[13] Kuznetsov S. A., Huang K. E., Marshall G. W., Robey P. G., Mankani M. H. (2008). Long-term stable canine mandibular augmentation using autologous bone marrow stromal cells andhydroxyapatite/tricalcium phosphate. *Biomaterials*.

[14] Almasri M., Altalibi M. (2011). Efficacy of reconstruction of alveolar bone using an alloplastic hydroxyapatite tricalcium phosphate graft under biodegradable chambers. *The British Journal of Oral & Maxillofacial Surgery*.

